# Frontiers and Hotspot Evolution of NLRP3 Inflammasome in Myocardial Infarction Research: A Bibliometric Analysis From 2013 to 2024

**DOI:** 10.1155/cdr/5178894

**Published:** 2025-02-19

**Authors:** Jin-wen Wu, Qi Lan, Ding-shan Zhang, Yu-hong Jian, Lin Yu, Rui Hao, Ping Liu, Gang Luo, Ming-tai Chen, Meng-nan Liu

**Affiliations:** ^1^Department of Cardiovascular Medicine, The Affiliated Traditional Chinese Medicine Hospital, Southwest Medical University, Luzhou, Sichuan, China; ^2^Department of Orthopaedics, West China Hospital, Sichuan University/West China School of Nursing, Sichuan University, Chengdu, Sichuan, China; ^3^School of Nursing, Southwest Medical University, Luzhou, Sichuan, China; ^4^Department of Cardiovascular Disease, Shenzhen Traditional Chinese Medicine Hospital, Shenzhen, Guangdong, China

**Keywords:** bibliometric analysis, myocardial infarction, myocardial ischemia–reperfusion injury, NLRP3

## Abstract

The NACHT, leucine-rich repeat, and pyrin domain–containing protein 3 (NLRP3) inflammasome plays an essential role in myocardial infarction (MI) development. Up to now, no bibliometric analyses of NLRP3 in MI have been performed. Publications related to NLRP3 in MI from 1 January 2013 to 20 August 2024 were extracted from the Web of Science Core Collection (WoSCC). HistCite Pro, CiteSpace, VOSviewer, Scimago Graphica, and bibliometric online analysis program were used for bibliometric analysis and visualization. The impact of publications was assessed using the total global citation score (TGCS). A total of 324 articles (284 articles and 40 reviews) were included. China has published the most in this field, followed by the United States. Harbin Medical University was the leading institution for research related to NLRP3 in MI. Professor Abbate A. from the United States has made significant achievements in this field. *International Immunopharmacology* was the most active journal and *Journal of Cardiovascular Pharmacology* was the most cited journal. This study systematically summarizes the research results of NLRP3 in MI over the past 12 years. NLRP3 in myocardial ischemia–reperfusion injury (MIRI) will become a hot research topic, and translational research on NLRP3 inhibitors in MIRI will benefit a greater number of patients.

## 1. Introduction

Myocardial infarction (MI) is the result of coronary artery occlusion, which leads to irreversible damage due to severe and persistent myocardial ischemia [1, 2], and is the leading cause of death worldwide. Even though a timely reperfusion procedure remains the most effective strategy for reducing ischemia injury, limiting infarct size, and improving clinical outcomes [3], it was recognized early on that reperfusion may paradoxically lead to exacerbated and accelerated injury in the myocardium, referred to as myocardial ischemia–reperfusion injury (MIRI). As soon as the blood flow and oxygen delivery are restored, a sterile inflammatory response is activated [4]. While this response is essential to guarantee the healing of wounded tissue, uncontrolled or exuberant inflammation becomes a mechanism of disease, which greatly enhances MI-derived damage [5]. Sterile inflammatory response is initiated by the detection of damage-associated molecular patterns (DAMPs) via intracellular pattern recognition receptors (PRRs) [6], and intracellular Nod-like receptors (NLRs) have been recognized to be significant mediators of inflammatory responses within these PRRs [7, 8].

The most studied inflammasome is NACHT, leucine-rich repeat (LRR), and pyrin domain (PYD)–containing protein 3 (NLRP3), a PRR that belongs to the NLR family [9]. NLRP3 is an intracellular guardian that can sense danger signals, such as tissue injury and microbial infection, leading to the induction of immune responses [10]. When activated by multiple stimuli, NLRP3 interacts homotypically with the adapter protein apoptosis-associated speck-like protein containing a caspase recruitment domain (ASC). It recruits caspase-1 and activates it to form intracellular multiprotein complexes called inflammasome [11, 12]. The NLRP3 inflammasome plays a crucial role in sterile inflammatory as a bridge to the immune inflammatory response. It has been shown that ischemia and reperfusion injury induce activation of NLRP3 inflammasome, leading to a second wave of inflammatory insults within minutes to hours of reperfusion [13]. Inhibition of the NLRP3 inflammasome pathway is thought to exert myocardial protection in the MIRI model [14]. These reports imply that the NLRP3 inflammasome may play a pivotal role in the pathophysiology of MI and may suggest that inhibition of the NLRP3 pathway could offer a viable therapeutic option. Given the active and promising research on the role of NLRP3 in MI, it is necessary to analyze the hotspots and trends in this field.

Bibliometrics is a sophisticated literature research methodology for quantitative and qualitative analysis of published papers. The analysis of NLRP3 in the field of MI, using literature visualization, enables a rapid understanding of the trends in the field. Targeting high-quality authors and high-productivity research organizations, research topics with credible and convincing high-quality literature can be quickly identified to accelerate the introduction process of scientific research. Although studies related to NLRP3 in MI have been reviewed from multiple perspectives currently, there is still a lack of visual analyses and summaries of their publication trends, main authors, and research hotspots. This paper analyzes the publications related to NLRP3 in MI using bibliometrics to visualize the distribution of annual publications, countries, institutions, authors, source journals, keywords, and cocitations in Web of Science (WoS) for 2013–2024. It is aimed at portraying the characteristics, status, research priorities, and trends of studies related to NLRP3 in MI and at providing insights that point to future research directions.

## 2. Materials and Methods

There are no animal or human studies presented in this manuscript, and there are no potentially identifiable human images or data. Therefore, ethical approval is not required for this work.

### 2.1. Data Collection

**Figure 1:**
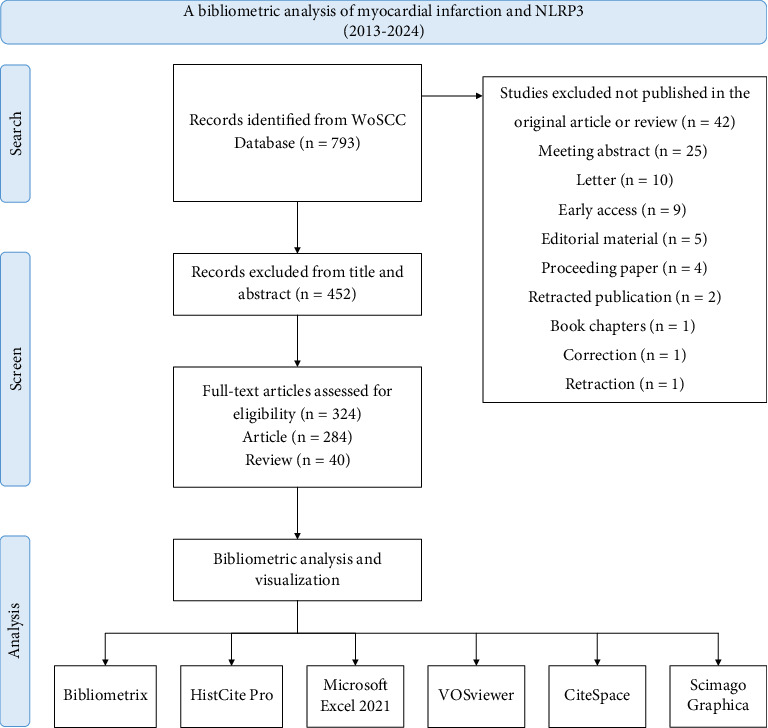
Flowchart of this bibliometric study.

The literature search was performed on the Web of Science Core Collection (WoSCC) on August 20, 2024. The search strategy employed was as follows: a combination of the following terms related to the NLRP3 inflammasome: “TS = [(NLRP3) OR (NLRP3 inflammasome) OR (NLR Family Pyrin Domain Containing 3 Protein) OR (Nod-like receptor protein 3 inflammasome)],” and terms related to MI: “TS = [(myocardial infarction) OR (Myocardial Infarctions) OR (Infarction, Myocardial) OR (Infarctions, Myocardial) OR (Myocardial Infarct) OR (MI)].” The search strategy is illustrated in Figure 1. Only articles and review articles published in English and meeting the specified inclusion criteria were considered for the analysis. The search process was conducted independently by two authors (Y.H.J. and R.H.), who ensured that all relevant studies were captured. All records, including references cited within the studies, were exported in plain text format for further analysis.

### 2.2. Data Analysis

In this study, HistCite Pro (version 2.1) was used to count records, total local citation scores (TLCSs), and total global citation scores (TGCSs) for each publication, journal, country, institution, and author.

VOSviewer (version 1.6.19) is a free software with powerful bibliometric mapping features [15]. In our study, the software mainly accomplished the following analyses: institutional collaboration, journal cocitation, keyword co-occurrence, author collaboration, author cocitation, reference cocitation, etc. The linkage between the nodes indicates the co-occurrence relationship, and the magnitude of the linkage indicates the frequency of co-occurrence of the two nodes.

CiteSpace (version 6.3.R1), another citation visualization and analysis tool developed by Professor Chen [16], was used for cluster analysis, co-occurrence analysis, and reference and keyword citation burst analysis.

Scimago Graphica (version 1.0.42) is a novel data visualization authoring tool that can be used for describing the global distribution map of academic research in this field and providing geographic distribution maps of publications.

In addition, the online bibliometrics website (https://bibliometric.com/app) was used to visualize international collaboration. Microsoft Excel 2021 was used to provide analysis of annual trends in the number of publications.

## 3. Results

A total of 324 publications (284 articles and 40 reviews) spanning a 12-year period (2013–2024) were included with a total of 10,549 citations.

### 3.1. Global Overview of Publication Output

**Figure 2:**
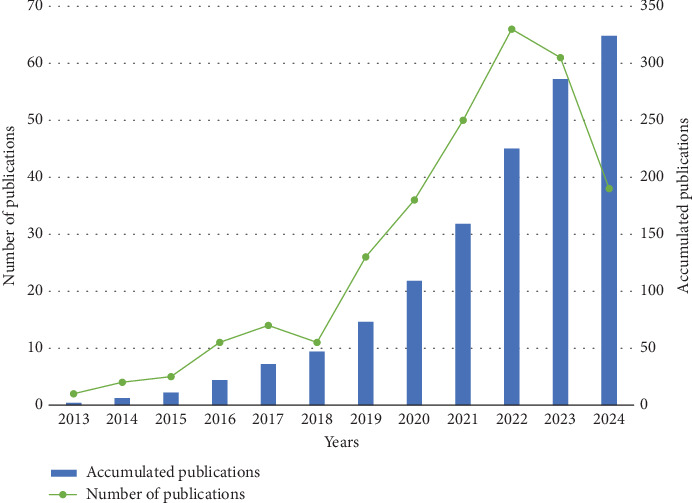
The annual number and the cumulative number of publications.

The number of studies on NLRP3 in MI has trended upward over the past 12 years, from 2 in 2013 to 61 in 2023, while the number of publications in 2024 was lower than that in 2023, probably because the search results included only two-thirds of the year 2024, as shown in Figure 2. Between 2013 and 2016, the number of annual publications was low, remaining at the single-digit level each year. However, since 2017, the number of annual publications has shown a clear trend of growth, particularly between 2019 and 2022, when the number of annual publications peaked, with nearly 70 publications in 2022. Publication output has grown rapidly, with the achievement of reaching a cumulative total of 324 publications in 2024.

**Figure 3:**
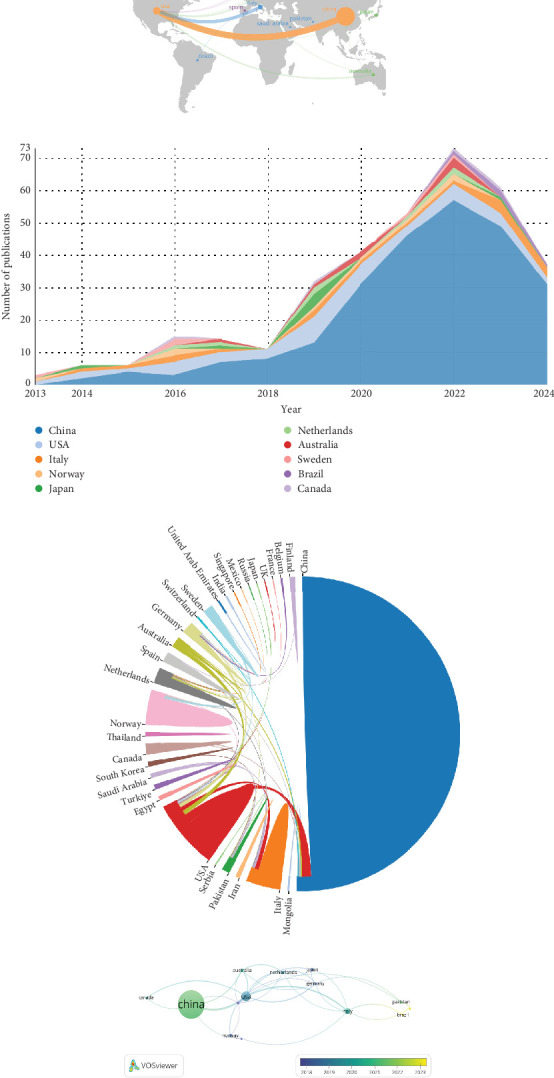
Visualization of countries. (a) The geographic distribution map based on the total literatures of different countries/regions. (b) Top 10 countries based on publication numbers every year. (c) The network visualization map of country coauthorship analysis. (d) The overlay visualization map of country coauthorship analysis. The node color reflected the corresponding average appearing year (AAY) according to the color gradient in the lower right corner.

The number of national publications was analyzed to investigate the countries/regions contributing most to the field. The analysis revealed that a total of 31 countries/regions have published in the relevant field, as shown in Figure 3(a), which showed the geographical distribution of publications, indicating that research is concentrated in Asia, North America, and Europe. The top-contributing countries were highlighted based on the number of publications (Figure 3(b), Table 1), where China ranked first with 251 (71.51%) publications, followed by the United States (*n* = 41, 11.68%), Italy (*n* = 17, 4.84%), Norway (*n* = 8, 2.28%), and Australia (*n* = 7, 1.99%). A visual map of national collaboration networks was constructed using online bibliometric websites, shown in Figure 3(c). China and the United States are the countries at the center of these publications and maintain strong collaborative relationships with several countries. Meanwhile, VOSviewer conducted an overlay visualization map of the country coauthorship analysis, with nodes colored concerning time, as shown in Figure 3(d). Countries labeled in green, such as China, Canada, and Italy, are relatively new researchers, while Norway, Spain, and Sweden are older researchers.

### 3.2. Analysis of Institutional Output and Collaboration

**Figure 4:**
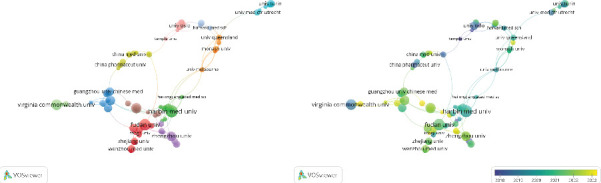
Visualization of institutions. (a) The network visualization map of journal coauthorship analysis. (b) The overlay visualization map of journal coauthorship analysis.

In general, a total of 416 institutions conducted research related to MI and NLRP3. The top 10 institutions are shown in Table 2. The majority of publications among these institutions were contributed by Harbin Medical University (*n* = 16), followed by Fudan University (*n* = 14), Virginia Commonwealth University (VCU) (*n* = 13), Wuhan University (*n* = 12), and China Academy of Chinese Medical Sciences (*n* = 10). VCU had the highest TGCS (1529 citations), followed by the University of Oslo (732 citations) and VCU Johnson Center for Critical Care and Pulmonary Research (676 citations). Coauthorship analysis was also conducted to investigate the collaborative relationships between institutions, with the size of the dots representing the number of articles published by the institutions and the color of the dots representing the clustering of the institutions, as shown in Figure 4(a). Harbin Medical University, Fudan University, and Zhengzhou University demonstrated high academic output in this research area and had extensive collaborations with other institutions. The red cluster was concentrated around Fudan University and contained the largest number of institutions (*n* = 12). By analyzing the coauthorship plus time overlap of the institutions, as shown in Figure 4(b), the institutions represented by University of Oslo and VCU were the early researchers in the field. While Harbin Medical University, Fudan University, and Zhengzhou University in China have been active in the field of NLRP3 and MI in recent years.

### 3.3. Analysis of Journals

**Figure 5:**
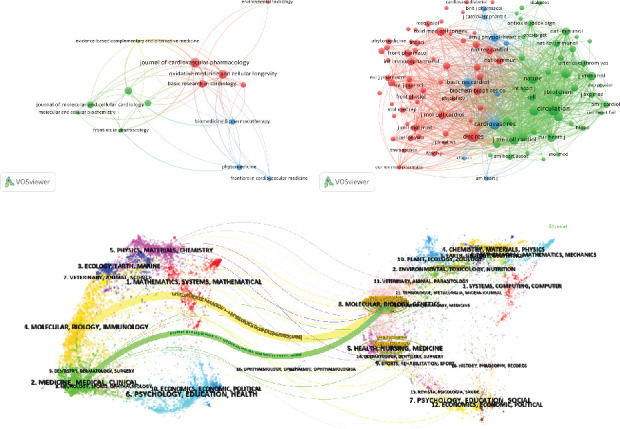
Visualization of journals. (a) The network visualization maps of citing journals. (b) The network visualization maps of cocited journals. (c) The dual-map overlay of journals from 2013 to 2024.

One hundred and sixty-nine journals were involved in the publication of articles on NLRP3 in MI.  Table 3 lists the top 10 journals based on the number of publications, with 25% of the publications being included. Among these, *International Immunopharmacology* (*n* = 14) published the most articles in this area, followed by *Journal of Cardiovascular Pharmacology* (*n* = 9) and *Journal of Cellular and Molecular Medicine* (*n* = 9). Among the top 10 active journals, *Oxidative Medicine and Cellular Longevity* had the highest impact factor (IF) (7.31) and *Journal of Cardiovascular Pharmacology* had the highest total citations (TGCS = 644) in Journal Citation Reports (JCR). Seventy percent of the journals were categorized as Q2 or Q3. In addition, VOSviewer was used to create a network visualization of citing and cocited journals, as shown in Figures 5(a) and 5(b). The minimum number of citations was 30, and a total of 109 cocited journals were included. *Circulation* had the highest number of cocitations, followed by *Circulation Research* and *Cardiovascular Research*. To illustrate the distribution of citing and cited journals, a double-map overlay was utilized, as depicted in Figure 5(c). The citing journals are represented by geographic regions on the left, while the cited journals are displayed on the right. Different line colors represent various disciplines. The citing journals are primarily associated with fields such as molecular, biology, and genetics, while the cited journals predominantly relate to molecular, biology, immunology, medicine, and clinical.

### 3.4. Analysis of Authors and Cocited Authors

**Figure 6:**
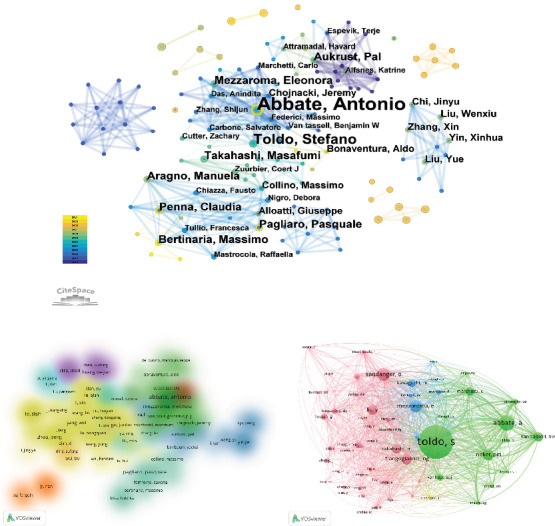
Visualization of authors. (a) The network visualization map of authors. (b) The cluster visualization map of author cooperation analysis. Authors with close collaborative relationships are assigned to the same cluster with the same color. (c) The network visualization map of cocited authors. The larger the node, the more citations were acquired.

A total of 2079 authors were involved in MI and NLRP3 studies. In Table 4, the most active author was Abbate A. (*n* = 14, TGCS = 2035), followed by Toldo S. (*n* = 12, TGCS = 1994), Pagliaro P (*n* = 8, TGCS = 293), Penna C (*n* = 7, TGCS = 189), Mezzaroma E. (*n* = 6, TGCS = 706). The authors' coauthorship network diagram is depicted in Figure 6(a), with color intensity indicating the time evolution of research collaborations. Abbate A. was an early researcher with a sustained interest in the field. The top 10 most cited authors are shown in Table 4. The five most cited authors were Abbate A. (TGCS = 2035), followed by Toldo S. (TGCS = 1994), Marchetti C. (TGCS = 998), van Tassell B.W. (TGCS = 738), and Mezzaroma E. (TGCS = 706). Cluster density maps for the author cocited analysis were depicted using VOSviewer (Figure 6(b)), with a minimum paper publication count of 2. A total of eight author clusters were formed. Sixty-one authors with at least 20 citations were included in the author cocited analysis, and the top three authors for total link strength were Toldo S. (5429), Abbate A. (3858), and van Tassell B.W. (2818).

### 3.5. Analysis of Publications and Cocited References

**Figure 7:**
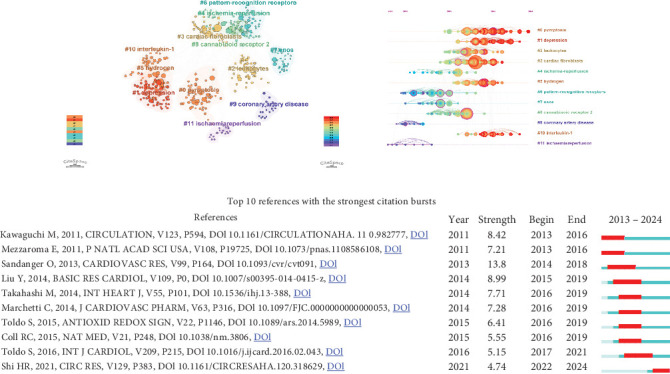
Visualization of references. (a) The cluster view map of reference cocitation analysis. (b) The timeline view map of reference cocitation analysis. (c) The top 10 references with the strongest citation bursts. The bars in blue represented the timeline; the bars in red represented a burst period of the references.

The most cited publications in a particular field reveal the impact of the research.  Table 5 characterized the top 10 cited publications, all with more than 200 citations. The most cited paper was “The NLRP3 Inflammasome in Acute Myocardial Infarction” by Toldo S. in *Nature Reviews Cardiology*. More than half of the articles were of article type. CiteSpace was used for clustering analysis of reference keywords and visualization of a timeline representation based on cocitation clustering, as shown in Figures 7(a) and 7(b). The clusters with different colors represent different research topics, which are divided into 11 clusters in total. According to the time of the cited references, the node size of the clustering reflects the research activity of the keyword in this period, and it can be seen that the research hotspots have been evolving and changing over time, and #11 ischaemia reperfusion and #9 coronary artery disease are the earlier research topics in the field, while #0 pyroptosis and #1 depression have shown strong continuity and activity in recent years. To better understand the evolution of MI and NLRP3-related research, the 20 most cited references on CiteSpace were also explored (Figure 7(c)). The first cited burst occurred in 2013 with an article published in 2011 by Kawaguchi M. et al. The article published by Sandanger O. et al. had the greatest burst intensity (13.8).

### 3.6. Analysis of Keywords and Themes

**Figure 8:**
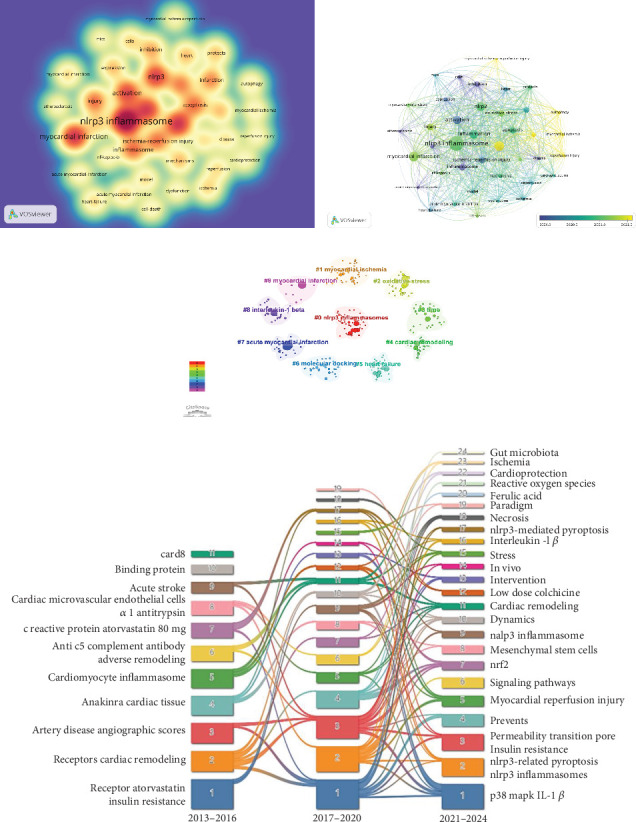
Visualization of keywords. (a) The cluster visualization map of keyword analysis. (b) The timeline view map of keyword analysis. (c) The cluster view map of keyword analysis. (d) Thematic evolution of the three stages in this field.

**Figure 9:**
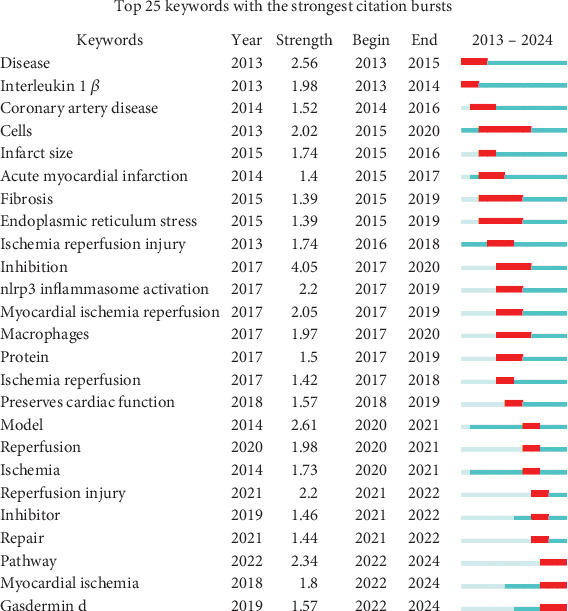
The top 25 keywords with the strongest citation bursts.

Trend assessment and analysis of keywords are important for revealing cutting-edge knowledge in a particular field. A total of 1310 keywords were collected in this study and keyword co-occurrence network analysis was performed using VOSviewer software as shown in Figure 8(a). In Table 6, the top 20 most frequently occurring keywords were summarized based on their frequency, with “NLRP3 inflammasome,” “myocardial infarction,” “pyroptosis,” “inflammation,” and “NLRP3” identified as the five most commonly used terms. To better elucidate the relationship between these keywords and the evolving trends in MI and NLRP3-related research, a visual mapping of 39 keywords with at least 15 occurrences is presented in Figure 8(b). In this mapping, dark blue indicates earlier occurrences, while yellow represents more recent occurrences. Keywords such as “inhibition,” “atherosclerosis,” and “inflammasome” were associated with earlier research, as indicated by their average time of occurrence. Notably, research on the NLRP3 inflammasome surged significantly after 2020, particularly in connection with emerging topics such as oxidative stress and autophagy. A keyword cluster analysis was also created via CiteSpace, and a total of 10 clusters emerged, as shown in Figure 8(c), including #0 NLRP3 inflammasomes, #1 myocardial ischemia, #2 oxidative stress, #3 time, #4 cardiac remodeling, #5 heart failure, #6 molecular docking, #7 acute myocardial infarction, #8 interleukin-1 beta, and #9 myocardial infarction. The modular *Q*-value and mean silhouette *S*-value were both greater than 0.5. In addition to quantitative analysis, this study also conducted a qualitative analysis of the keywords.  Figure 8(d) illustrates the evolution of keywords across three phases of the field from 2013 to 2024, summarized using a Sankey diagram. In this diagram, the bandwidth proportionally represents the frequency of occurrences, with wider bands indicating higher frequency.  Figure 9 highlights the top 25 keywords with the most significant citation bursts in the field, with “inhibition,” “pathway,” “reperfusion injury,” and “NLRP3 inflammasome activation” showing the highest burst intensities. Notably, “pathway,” “gasdermin D,” and “myocardial ischemia” are keywords that continue to exhibit citation bursts through 2024.

## 4. Discussion

### 4.1. General Information

In this study, research data on the NLRP3 inflammasome in MI were retrieved from the WoSCC database and analyzed using bibliometric methods to explore the research trajectory and trends in this field. The analysis encompassed trends in publications, major contributing countries, institutions, journals, authors, references, and keywords. A total of 324 articles and reviews published between 2013 and 2024 were retrieved. The first article, published in 2013 by Hermansson et al. [17], suggested that the NLRP3 inflammasome might be associated with exacerbating ischemic myocardial injury. Based on the annual publication count and growth trends, the research can be divided into two phases: a slow-growth phase from 2013 to 2018, with fewer than 15 articles published per year, and a fast-growth phase from 2019 to 2024, with more than 25 publications per year. This trend indicates that interest in the NLRP3 inflammasome within the MI research community is likely to remain strong in the coming years.

### 4.2. Country and Institution

In terms of countries and regions studied, China had the highest number of publications, followed by the United States. However, in terms of TLCS, the United States was the most cited country, with China in second place, indicating that both countries have had a significant presence in the field over the past 12 years. The visualization map of intercountry cooperation networks shows that China and the United States dominate the global collaboration network, while other countries' cooperation tends to be more regionally focused. These intercountry cooperation networks not only reflect partnerships in scientific research but also reveal the influence of various factors, such as politics and economics, on research collaboration. Although China entered this field later than some other countries, interest has grown rapidly in recent years, leading to a steady increase in the number of published articles. National economic support and international cooperation are likely to further promote the overall development of this field. By analyzing the field from different perspectives, researchers can gain a more comprehensive understanding of which countries are currently leading and should be prioritized for collaboration, particularly China, the United States, and Italy.

Given that China had the highest total number of publications, it is unsurprising that nearly all of the top 10 institutions were from China, underscoring the country's strong research capabilities. Harbin Medical University, in particular, has made a significant contribution to this field. Notably, while the University of Oslo in Norway ranks 10th in terms of total publications, it ranks second among the top 10 institutions in terms of citations. This discrepancy may be attributed to the university's early involvement in the field and its extensive original research, which has been highly regarded for its depth and quality. Although Harbin Medical University is the only Chinese institution among the top 10 most-cited institutions, Fudan University, Guangzhou University of Chinese Medicine, and Zhengzhou University have been the most active in the last 2 years. International academic exchanges and collaborations are also on the rise; however, collaborations involving Chinese institutions are primarily domestic. It is advisable to encourage greater partnerships between Chinese institutions and their global counterparts.

### 4.3. Journal

Among the top 10 journals, 60% fall within JCR Q2 or Q3, and they were categorized under immunology, pharmacology, molecular and cellular biology, alternative medicine, and cardiology. Notably, only one journal, *International Immunopharmacology*, has published more than 10 articles on MI and NLRP3 research, indicating that this field still requires substantial further investigation. Moreover, although China has made significant contributions to this research area, most publications are still disseminated through journals managed by Western countries. This suggests that Asian countries, including China, could enhance their efforts in developing journals dedicated to this field. Furthermore, the cocitation of journals like *Circulation* and *Cardiovascular Research* indicates that the most impactful studies in this field are often interdisciplinary, combining insights from cardiology, immunology, and molecular biology. Future research could benefit from a similar multidisciplinary approach, integrating these domains to develop more comprehensive therapeutic strategies against MI by targeting the NLRP3 inflammasome pathway.

### 4.4. Author

In the co-occurrence analysis of authors, Professor Abbate A. from VCU emerges as a leading figure in NLRP3-related research in the context of MI. He has not only produced the highest number of publications but is also among the earliest researchers in this domain. Professor Abbate's research primarily focuses on the role of the inflammasome in ischemic MI, particularly the NLRP3 inflammasome [18, 19]. His 14 publications in this field have been cited 2035 times, underscoring his significant contributions and the recognition he has garnered within the scientific community.

Since 2014, Professor Abbate has consistently explored the therapeutic potential of inhibiting inflammasome activity via NLRP3 to reduce infarct size following myocardial ischemia/reperfusion (I/R) in mice [18]. His research has since concentrated on identifying and testing inhibitors of the NLRP3 inflammasome, contributing valuable insights to the field [20]. Furthermore, Professor Abbate has closely collaborated with Toldo S., with their joint research extending through 2024 [21]. Both authors have made substantial contributions to the study of MI and NLRP3, and their work is poised to continue influencing the field in the years to come.

### 4.5. Reference

Among the 10 most cited articles, the most frequently cited is a review by Toldo and Abbate [19], published in *Nature Reviews Cardiology* in 2015, which focuses on the role of the NLRP3 inflammasome in acute MI. The second most cited article, authored by Abbate et al. [22] and published in 2020, systematically summarizes the evidence supporting the association between the NLRP3 inflammasome and IL-1 cytokines with the pathogenesis of cardiovascular disease. Citation burst analysis, which indicates the extent to which a reference is rapidly gaining attention and being widely discussed, reflects trends in the popularity of particular research areas. The first significant citation burst originated from a 2011 article by Kawaguchi et al. [23], which elucidated the molecular basis of the initial inflammatory response after I/R and proposed the inflammasome as a novel therapeutic target for the prevention of MIRI. This study provided a foundational direction for subsequent research on MI and the NLRP3 inflammasome. Additionally, a 2013 publication by Sandanger et al. [24] demonstrated the highest burst intensity. In this study, they observed that NLRP3-deficient mice undergoing postischemic reperfusion of the heart showed significant improvements in cardiac function and reductions in hypoxic injury compared to wild-type mice.

### 4.6. Research Hotpots

Through the analysis of reference timelines, keyword clustering, keyword bursts, and qualitative keyword analysis, the current research frontiers and hotspots can be broadly categorized into the following three aspects.

#### 4.6.1. Response Mechanisms of NLRP3 in MI

An increasing number of studies have identified NLRP3 as an initial sensor of the sterile inflammatory response in the pathophysiology of MI. During MI, NLRP3 is activated in response to noninfectious stimuli, such as cellular debris, and plays a crucial role in regulating the activation of caspase-1, as well as the production and secretion of potent proinflammatory cytokines, including IL-1*β* and IL-18 [25]. The activation of caspase-1 can induce a specific type of cell death known as pyroptosis. In this process, caspase-1 cleaves gasdermin D (GSDMD), a key effector protein in pyroptosis, facilitating the release of extracellular IL-1*β* and IL-18, which exacerbates myocardial injury. Further research has revealed that the NLRP3 inflammasome exhibits distinct actions and response mechanisms in different cardiac cell types. For instance, in monocytes, fibroblasts, and endothelial cells, activation of the NLRP3 inflammasome results in the release of large amounts of IL-1*β* [26, 27]. In contrast, although the NLRP3 inflammasome can form in cardiomyocytes, it releases only minimal amounts of IL-1*β* [28]. Notably, Marchetti et al. [29] demonstrated that in cardiomyocytes, the activation of caspase-1 leads to the secretion of IL-18 and localized cell death through pyroptosis. These findings highlight the involvement of the NLRP3 inflammasome in the inflammatory response associated with MI. However, further studies are required to deepen our understanding of how the NLRP3 inflammasome is activated and how it contributes to cellular pyroptosis in this context.

#### 4.6.2. Role of NLRP3 Inflammasome in MIRI

Reperfusion therapy, while essential for restoring blood flow after MI, can paradoxically induce further myocardial injury. The activation of the NLRP3 inflammasome in damaged cardiomyocytes has been closely linked to inflammatory injury. In I/R mouse cardiomyocytes, mitochondrial fission promotes the production of reactive oxygen species (ROS), leading to oxidative stress and subsequent activation of the NLRP3 inflammasome [30]. The pathophysiological role of the NLRP3 inflammasome has also been highlighted by Sandanger et al. [24], who demonstrated that in a mouse model of MI, NLRP3 inflammasome expression was predominantly upregulated in cardiac fibroblasts within ischemic myocardium. Their study further revealed that cardiac fibroblasts secrete IL-1*β* in response to extracellular ATP, which is released during MI as a result of myocardial injury. Notably, reduced MIRI was observed in NLRP3-knockout (KO) mice, but not in ASC-KO mice, suggesting that NLRP3 and ASC may have distinct roles in myocardial injury following I/R. Similarly, Shigeoka et al. [31] found that renal I/R injury was attenuated in NLRP3-KO mice, whereas ASC-KO mice did not exhibit such attenuation. These findings suggest that inflammasome components like NLRP3 and ASC may function independently in MIRI.

#### 4.6.3. NLRP3 Inhibitors May Be New Targets for the Prevention and/or Treatment of MI and MIRI

Studies have focused on reducing I/R injury by inhibiting signaling pathways or directly inhibiting NLRP3 activation to protect cardiomyocytes. Preclinical studies in transgenic mouse models and the use of targeted inhibitors have shown that inhibition of NLRP3 inflammasome activation reduces inflammatory injury and adverse cardiac remodeling [26]. Colchicine, an ancient anti-inflammatory agent recently found to inhibit the assembly and activation of NLRP3, has been shown to significantly inhibit, in a mouse model of acute myocardial ischemia, the 24-h increase in inflammasome activity afterward [32]. Inhibitors targeting NLRP3 inflammasome as well as blockers of IL-8 and IL-6 (downstream of IL-1) are being clinically tested against a variety of cardiovascular diseases, and the discovery by Marchetti et al. [18] of a novel inhibitor derived from glyburide, a molecule that fails to induce insulin release, which significantly reduces the size of infarcts and protects cardiac function, also demonstrates that early NLRP3 inflammasome activation is critical for downstream inflammatory signaling. In a recent study [33], it was noted that cinnamamide derivative compound 7 significantly improved left ventricular systolic function, infarct size, and cardiac apoptosis after MIRI. This study confirmed that cinnamamide derivative compound 7 attenuated myocardial injury by inhibiting the NLRP3 pathway. Such studies are often combined with molecular docking techniques to find potential NLRP3 inhibitors.

## 5. Limitations

It is important to acknowledge the limitations of this study. Firstly, as a bibliometric analysis, the data collection and processing are heavily reliant on software tools, which cannot fully replace the comprehensiveness of systematic searches. Secondly, this study exclusively utilized data from WoSCC, omitting other databases such as Scopus, which may have resulted in data gaps. Additionally, our analysis was confined to English-language articles, introducing a potential selection bias. Lastly, due to the rapid evolution of databases, some of the most recent studies might not have been captured in our analysis.

## 6. Conclusion

In summary, this study presented a bibliometric analysis of NLRP3 research in the context of MI using tools such as HistCite Pro, CiteSpace, VOSviewer, and Scimago Graphica. The findings indicated that research in this area has experienced rapid growth from 2013 to 2024. China and the United States have emerged as the leading contributors, highlighting the importance of enhanced international collaboration. Recent research trends have focused on the mechanisms of NLRP3 activation in MI, the role of NLRP3 in MIRI, and the therapeutic potential of NLRP3 inhibitors. These studies are likely to pave the way for the discovery of novel drugs and therapeutic targets, thereby improving clinical outcomes by offering innovative strategies and insights.

## Figures and Tables

**Table 1:** Top 10 active countries/regions.

**Rank**	**Country/region**	**Publications**	**Percent**	**TLCS**	**TGCS**
1st	China	251	71.51%	300	5948
2nd	United States	41	11.68%	493	4095
3rd	Italy	17	4.84%	106	798
4th	Norway	8	2.28%	121	740
5th	Australia	7	1.99%	7	623
6th	Netherlands	7	1.99%	40	557
7th	Japan	7	1.99%	102	478
8th	Sweden	5	1.42%	3	360
9th	Germany	4	1.14%	23	300
10th	Canada	4	1.14%	2	203

**Table 2:** Top 10 most active institutions and cited institutions.

**Rank**	**Most active institutions**				**Most cited institutions**			
	**Institutions**	**Publications**	**TLCS**	**TGCS**	**Institutions**	**Publications**	**TLCS**	**TGCS**
1st	Harbin Med Univ	16	42	520	Virginia Commonwealth Univ	13	219	1529
2nd	Fudan Univ	14	9	329	Univ Oslo	8	119	732
3rd	Virginia Commonwealth Univ	13	219	1529	VCU Johnson Ctr Crit Care & Pulm Res	2	120	676
4th	Wuhan Univ	12	1	363	Norwegian Univ Sci & Technol	3	93	622
5th	China Acad Chinese Med Sci Sciences	10	2	172	Harbin Med Univ	16	42	520
6th	Zhengzhou Univ	10	8	110	Univ Queensland	4	6	508
7th	Guangzhou Univ Chinese Med	8	19	134	Div Cardiothorac Surg	1	78	429
8th	Nanjing Med Univ	8	6	78	Pauley Heart Ctr	1	78	429
9th	Nanjing Univ Chinese Med	8	4	151	Sch Pharm	1	21	393
10th	Univ Oslo	8	119	732	Univ Colorado Denver	1	21	393

**Table 3:** Top 10 most active journals.

**Rank**	**Journals**	**Country**	**Publications**	**IF (2023)**	**JCR (2023)**	**TGCS**
1st	*International Immunopharmacology*	Netherlands	14	4.8	Q2	319
2nd	*Journal of Cardiovascular Pharmacology*	United States	9	2.6	Q2	644
3rd	*Journal of Cellular and Molecular Medicine*	England	9	4.3	Q2	222
4th	*Oxidative Medicine and Cellular Longevity*	United States	8	7.31	Q2	545
5th	*Evidence-based Complementary and Alternative Medicine*	England	8	2.65	Q3	58
6th	*Biomedicine & Pharmacotherapy*	France	7	6.9	Q1	182
7th	*Phytomedicine*	Germany	7	6.7	Q1	209
8th	*Journal of Molecular and Cellular Cardiology*	England	7	4.9	Q1	295
9th	*Biochemical and Biophysical Research Communications*	United States	6	2.5	Q3	171
10th	*Molecular and Cellular Biochemistry*	Netherlands	6	3.5	Q3	76

**Table 4:** Top 10 most active authors and cited authors.

**Rank**	**Most active authors**			**Most cited authors**		
	**Author**	**Publications**	**TGCS**	**Cocited author**	**Publications**	**TGCS**
1st	Abbate A.	14	2035	Abbate A.	14	2035
2nd	Toldo S.	12	1994	Toldo S.	12	1994
3rd	Pagliaro P.	8	293	Marchetti C.	5	998
4th	Penna C.	7	189	van Tassell B.W.	3	738
5th	Mezzaroma E.	6	706	Mezzaroma E.	6	706
6th	Zhou P.	6	49	Aukrust P.	4	649
7th	Ge J.B.	6	244	Yndestad A.	4	649
8th	Marchetti C.	5	998	Ranheim T.	3	627
9th	Zhang M.	5	39	Zhang S.J.	4	613
10th	Li X.	5	145	Mauro A.G.	4	582

**Table 5:** Top 10 highly cited publications.

**Rank**	**Title**	**First author**	**Year**	**Type**	**TGCS**	**Journal**	**IF (2023)**
1st	The NLRP3 Inflammasome in Acute Myocardial Infarction	Toldo S.	2018	Review	437	*Nature Reviews Cardiology*	41.7
2nd	Interleukin-1 and the Inflammasome as Therapeutic Targets in Cardiovascular Disease	Abbate A.	2020	Article	396	*Circulation Research*	16.5
3rd	The NLRP3 Inflammasome is Up-Regulated in Cardiac Fibroblasts and Mediates Myocardial Ischaemia/Reperfusion Injury	Sandanger O.	2013	Article	390	*Cardiovascular Research*	10.2
4th	NLRP3 Inflammasome Activation-Mediated Pyroptosis Aggravates Myocardial Ischemia/Reperfusion Injury in Diabetic Rats	Qiu Z.	2017	Article	277	*Oxidative Medicine and Cellular Longevity*	7.31
5th	The Selective NLRP3-Inflammasome Inhibitor MCC950 Reduces Infarct Size and Preserves Cardiac Function in a Pig Model of Myocardial Infarction	van Hout G.P.J.	2017	Article	254	*European Heart Journal*	37.6
6th	TXNIP Mediates NLRP3 Inflammasome Activation in Cardiac Microvascular Endothelial Cells as a Novel Mechanism in Myocardial Ischemia/Reperfusion Injury	Liu Y.	2014	Article	250	*Basic Research in Cardiology*	7.5
7th	Inflammasome, Pyroptosis, and Cytokines in Myocardial Ischemia-Reperfusion Injury	Toldo S.	2018	Review	248	*American Journal of Physiology-Heart and Circulatory Physiology*	4.1
8th	Signaling Pathways and Targeted Therapy for Myocardial Infarction	Zhang Q.	2022	Review	235	*Signal Transduction and Targeted Therapy*	40.8
9th	NLRP3 Inflammasome Expression and Activation in Human Atherosclerosis	Varghese G.P.	2016	Article	217	*Journal of the American Heart Association*	5.0
10th	A Novel Pharmacologic Inhibitor of the NLRP3 Inflammasome Limits Myocardial Injury After Ischemia-Reperfusion in the Mouse	Marchetti C.	2014	Review	212	*Journal of Cardiovascular Pharmacology*	2.6

**Table 6:** Top 20 keywords.

**Rank**	**Keyword**	**Occurrence**
1st	NLRP3 inflammasome	78
2nd	Ischemia-reperfusion injury	78
3rd	Myocardial infarction	78
4th	Pyroptosis	70
5th	Inflammation	70
6th	NLRP3	66
7th	Activation	50
8th	Inflammasome	49
9th	Injury	48
10th	Apoptosis	78
11th	Oxidative stress	44
12th	Infarction	43
13th	Inhibition	35
14th	Expression	31
15th	Heart	31
16th	Mechanisms	31
17th	Protects	29
18th	Ischemia-reperfusion	26
19th	Cells	25
20th	Ischemia	25

## Data Availability

The data are available on request from the authors.
